# A Clinical Exploration of One of the Longest Documented Ventricular Pauses in an Ambulatory Patient

**DOI:** 10.1155/cric/9163647

**Published:** 2026-10-05

**Authors:** Alyaa Saleh, Lanah Almatroud, Jibran Ajaz Sheikh, Vipin Khetarpal

**Affiliations:** ^1^ Central Michigan University College of Medicine Saginaw Michigan USA cmich.edu; ^2^ Michigan State University East Lansing Michigan USA msu.edu

**Keywords:** arrhythmia, medical education, ventricular pause

## Abstract

Cardiac conduction system abnormalities, such as sinus nodal dysfunction and AV block, can lead to significant bradyarrhythmias and pauses, potentially causing symptoms like dizziness and loss of consciousness. Monitoring these rhythm disturbances is crucial, as they may evolve and indicate underlying structural heart disease or lead to more serious arrhythmias. We present the case of a 76‐year‐old man with an exceptionally prolonged 62‐second ventricular pause, followed by a single ventricular beat and an additional 24‐second pause before normal rhythm resumes. He presented recurrent, intermittent, and unpredictable episodes of dizziness that were not associated with postural changes. A resting electrocardiogram (ECG) showing a normal sinus rhythm with first‐degree heart block prompting placement of the event monitor and that detected the ventricular pauses. To our knowledge, our case represents one of the longest documented ventricular pauses in an ambulatory patient, emphasizing the need for ongoing evaluation in similar cases and the potential benefits of earlier intervention to prevent serious complications and death.

## 1. Introduction

The sinoatrial (SA) node and atrioventricular (AV) node play a crucial role in maintaining normal cardiac rhythm. Dysfunction can result in significant bradyarrhythmias and conduction pauses [1]. Although short sinus pauses of less than 2 s are generally considered benign [2], those lasting longer than 3 s can pose serious clinical concerns, particularly when occurring in symptomatic patients. Prolonged pauses and conduction system disturbances can lead to dizziness, syncope, and in extreme cases, heart failure or sudden cardiac death [3].

The incidence of sinus node dysfunction and AV block increases with age [1], making these conditions an important consideration in the management of elderly patients. In addition to aging, other common etiologies contributing to AV node dysfunction include ischemic heart disease, autonomic dysfunction, and medications such as beta and calcium channel blockers [4]. Understanding and monitoring these rhythm disturbances is vital, as they may not only indicate underlying structural heart disease but also progress into more severe arrhythmias, such as ventricular asystole, which can have life‐threatening consequences [5]. Early detection and timely intervention are crucial for enhancing patient outcomes and preventing complications.

Despite the prevalence of bradyarrhythmias, cases involving prolonged ventricular pauses exceeding 30 s are rarely documented, particularly in ambulatory patients. Most reported cases of significant ventricular pauses occur in inpatient settings [6, 7], where continuous telemetry allows for real‐time detection and management of these arrhythmias. In contrast, ambulatory cases pose unique diagnostic challenges, as these pauses often go unnoticed until symptoms prompt further investigation through extended monitoring techniques such as event recorders or Holter monitors. Such cases illustrate the importance of comprehensive diagnostic evaluation and timely management to mitigate the risks associated with these arrhythmias. We present the case of a 76‐year‐old male with an exceptionally long ventricular pause of 62 s, highlighting the clinical manifestations, diagnostic challenges, and therapeutic interventions for severe conduction system abnormalities.

## 2. Case Presentation

A 76‐year‐old male with a history of single‐vessel right coronary artery disease (status post drug‐eluting stent placement), Type 2 diabetes mellitus, hypertensive heart disease, and prostate cancer presented to a veterans affairs emergency department with recurrent, intermittent, and unpredictable episodes of dizziness that were not associated with postural changes. These episodes were not accompanied by vertigo, palpitations, dyspnea, wheezing, or chest pain. Upon initial presentation, his heart rate was 74 bpm, blood pressure was 129/82 mmHg, respiratory rate 17 bpm, and SpO2 was 96% on room air, with normal orthostatic vital signs. Cardiac, respiratory, and neurological examinations were unremarkable. A resting electrocardiogram (ECG) showed a normal sinus rhythm with first‐degree heart block.

An echocardiogram showed normal left ventricular size and systolic function (ejection fraction of 65%–70%), mild concentric left ventricular hypertrophy, Grade 1 diastolic dysfunction, and mild to moderate left atrial dilatation. There was no significant valvular dysfunction. A 72‐h ambulatory ECG revealed a minimum heart rate of 38 bpm, an average heart rate of 74 bpm, with significant findings including 9 h and 53 min of second‐degree AV block (Mobitz Type II) and patient‐triggered episodes temporally corresponding to the high‐grade AV blocks.

Given the abnormal ambulatory ECG findings, a 14‐day event monitor was ordered while evaluating for reversible causes of heart block simultaneously. The event monitor was placed 29 days following the 72‐h ambulatory ECG monitor. The monitor revealed sinus rhythm as the predominant rhythm but also detected significant abnormalities, including one episode of nonsustained ventricular tachycardia (lasting five beats), one episode of supraventricular tachycardia (lasting four beats), and five ventricular pauses, the longest being 62.9 and 24 s. These pauses were related to intermittent complete heart block (Figure 1 and 2). Analysis of thyroid function tests, electrolytes, and detailed medication review was unremarkable in evaluating for reversible etiologies.

**Figure 1:**
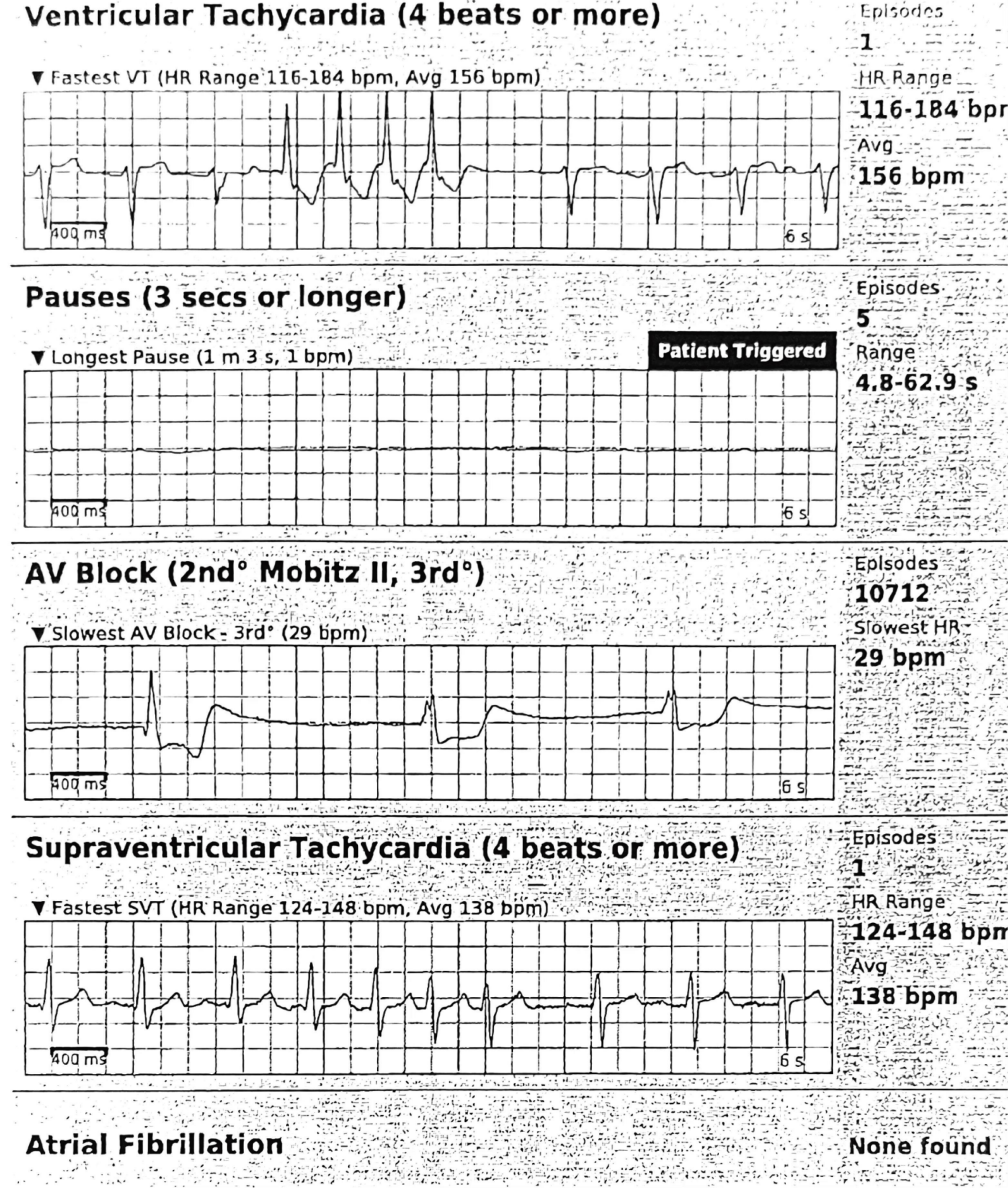
The 14‐day event monitor – Zio patch report key tracings.

**Figure 2:**
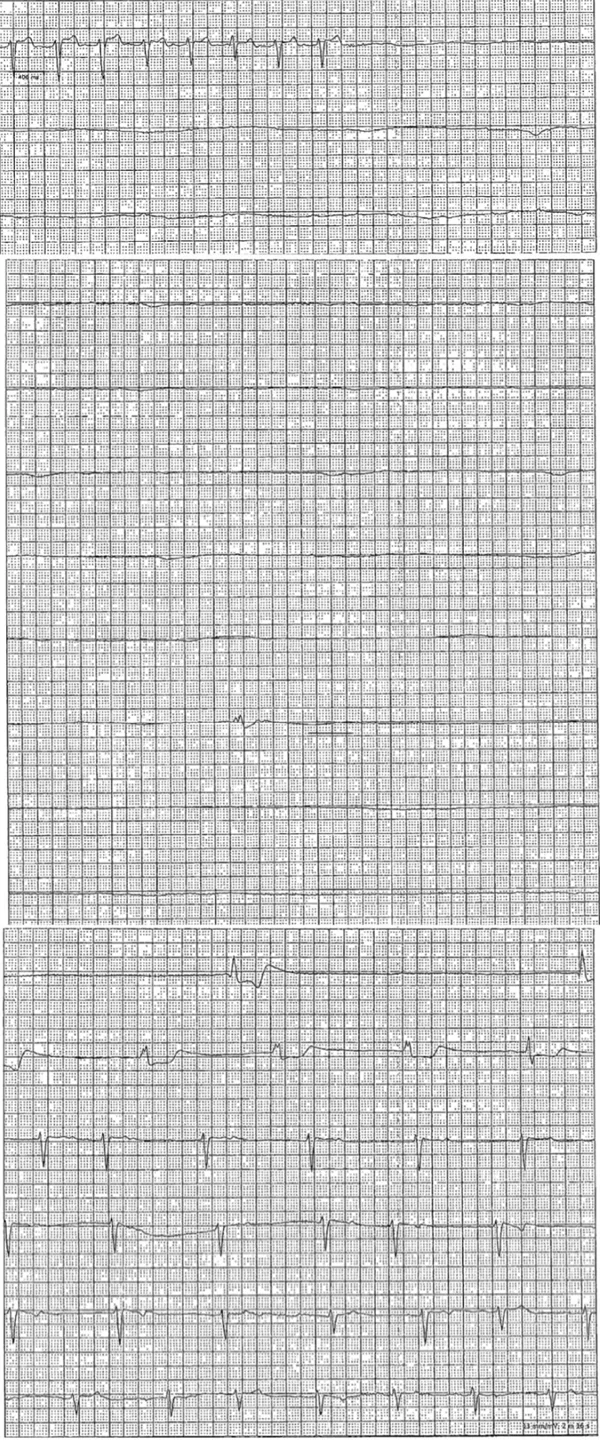
The 14‐day event monitor – A 62.9‐s ventricular asystole followed by a singular ventricular beat, followed by an additional 24‐s pause before the resumption of a regular rhythm.

During the 14‐day monitoring time, the patient experienced a syncopal episode corresponding to the 1‐min ventricular pause, during which his wife observed him lose consciousness briefly with his eyes rolling back and his skin turning blue. He also reported several other episodes of lightheadedness without loss of consciousness during this time. Despite these symptoms, he did not seek medical attention and continued with his daily activities. He was only evaluated in the hospital after being contacted by his cardiologist′s office following a review of the event monitor recordings.

In the hospital, the patient underwent uneventful placement of a permanent pacemaker for intermittent complete heart block causing ventricular asystole. At both his 1‐month and 3‐month follow‐up visits, he reported feeling well and was asymptomatic, with no further episodes of dizziness, syncope, or chest pain.

Overall, the clinical course spanned for over 3‐months from the initial presentation of intermittent dizziness to identification and permanent pacemaker implantation. The patient initially presented with recurrent episodes of dizziness and underwent a 72‐h ambulatory ECG. A 14‐day event monitor was then ordered to further evaluate the findings and was placed 29 days after completion of the initial ambulatory monitor. During the subsequent monitoring period, the patient experienced multiple ventricular pauses. Although he did not seek immediate medical attention following the syncopal episode, the event was later identified during review of the monitor by his cardiology team, prompting hospital evaluation and permanent pacemaker implantation. Following pacing, his dizziness and syncope resolved during his follow up‐evaluations. Written informed consent was obtained from the patient for publication of this case report.

## 3. Discussion

Ventricular pauses are significant arrhythmic events that can be exacerbated by increased vagal tone, often associated with rapid eye movement (REM) sleep [8]. During this phase of sleep, the parasympathetic nervous system predominates, increasing vagal tone, which can cause bradyarrhythmias even in patients without underlying cardiovascular disease [9]. Sampognaro et al. presented a case of a 63‐year‐old male who experienced dizziness after running or belching. A 14‐day Zio XT monitor revealed 971 sinus pauses lasting over 3 s, primarily occurring between 10 p.m. and 6 a.m., with the longest pause lasting 12.1 s [9]. Although the episodes in their patient′s case were nocturnal, our patient experienced a significantly prolonged ventricular pause of 62 s during waking hours, leading to syncope. This contrast highlights the varied presentation of conduction pauses in different clinical contexts, emphasizing that the duration, timing, and symptoms associated with these pauses can vary widely, requiring individualized evaluation and management.

The clinical symptoms and manifestations of conduction anomalies can vary based on the duration and frequency of the pauses as well as the patient′s medical history, potentially leading to misdiagnosis. Rathore et al. describe a case of sick sinus syndrome (SSS) and asystole mimicking a transient ischemic attack (TIA). A 75‐year‐old man with a history of TIA, dyslipidemia, and hypertension presented to the emergency department with recurring episodes of acute mental status changes, agonal breathing, and aphasia. After ruling out cerebrovascular causes and discovering significant bradycardia, further cardiac and electrophysiologic evaluations revealed three sinus pauses, the longest duration over 20 s. A leadless permanent pacemaker was implanted for his SSS, following which no recurrence of his symptoms occurred [7]. This case illustrates the importance of maintaining a high index of suspicion for cardiac etiologies in patients presenting with mental status changes, hypoperfusion, or symptoms resembling TIA, even when their medical history suggests alternative diagnoses. Comprehensive cardiac testing should be prioritized in such cases to ensure timely and accurate diagnosis.

In our patient, the prolonged symptomatic ventricular pauses (including the 62‐second ventricular arrest), caused by episodes of complete heart block and high‐grade AV block, illustrate the importance of timely and thorough evaluation, as they can lead to significant clinical consequences. These findings suggest a possible underlying pathology involving scarring or fibrosis of the conduction system, a phenomenon often seen in elderly patients or those with ischemic heart disease [10]. Age‐related degenerative changes to the conduction system, such as fibrosis of the SA and AV nodes, may contribute to the development of SSS and AV block. These arrhythmias can result in symptoms such as dizziness, syncope, and, in severe cases, sudden cardiac death if left untreated [3].

The patient′s symptoms, including syncope and lightheadedness, were directly attributable to the documented high‐grade AV block. Given the significant clinical manifestations and the risk of future arrhythmic events, permanent pacemaker implantation was the appropriate intervention. The pacing device addresses the bradycardia and the high‐grade AV block, ensuring a consistent heart rate and preventing further episodes of syncope. The decision to implant a pacemaker is consistent with current guidelines, which recommend pacemaker implantation in patients with symptomatic bradycardia, especially when accompanied by high‐degree AV block or SSS [11]. The improvement in the patient′s quality of life postimplantation, with the cessation of dizziness and loss of consciousness, further reinforces the benefits of early intervention in such cases.

It is also important to emphasize that not all patients with sinus bradycardia, pauses, or AV block require a pacemaker. Pacing is indicated and improves outcomes when conduction abnormalities are symptomatic and/or advanced, particularly in cases of AV block. In some instances, the disease process may be less severe, and conservative management with regular follow‐up may be appropriate [11]. The American Heart Association (AHA), American College of Cardiology (ACC), and Heart Rhythm Society (HRS) guidelines recommend monitoring patients with milder forms of cardiac electrical system diseases [11]. This highlights the need for personalized care, where clinical judgment plays a pivotal role in deciding the appropriate follow‐up strategy. Regular assessment via ambulatory ECG monitoring or event monitors may be beneficial in patients at risk for bradyarrhythmic events but without immediate indications for pacing. Commercially available wearable devices, such as smartwatches, may also play an evolving role in the monitoring of these arrhythmias.

## 4. Conclusion

To our knowledge, this case represents one of the longest ventricular asystole documented in an ambulatory patient, with the pause lasting 62.9 s. Although there are other reports of prolonged ventricular pauses, including some documented during sleep, the length and symptomatic nature of this patient′s pause during wakefulness, and physical activity are noteworthy. These findings highlight the need for continuous monitoring and timely intervention in similar high‐risk cases.

## Funding

No funding was received for this manuscript.

## Disclosure

The authors have nothing to report.

## Conflicts of Interest

The authors declare no conflicts of interest.

## Data Availability

Data sharing is not applicable to this article as no datasets were generated or analyzed during the current study.
